# Measurement of preprandial and postprandial urine calcium to creatinine ratios in male Miniature Schnauzers with and without urolithiasis

**DOI:** 10.1111/jvim.15690

**Published:** 2020-01-11

**Authors:** Susan V. Carr, David C. Grant, Stefanie M. DeMonaco, Megan Shepherd

**Affiliations:** ^1^ Department of Small Animal Clinical Sciences Virginia‐Maryland College of Veterinary Medicine, Virginia Tech Blacksburg Virginia; ^2^ Department of Large Animal Clinical Sciences Virginia‐Maryland College of Veterinary Medicine, Virginia Tech Blacksburg Virginia

**Keywords:** calciuresis, cystolithiasis, nephrolithiasis, veterinary

## Abstract

**Background:**

We aimed to identify a simple test for excessive calciuresis and predict calcium oxalate (CaOx) disease in Miniature Schnauzers. We investigated the impact of postprandial time on the urine calcium to creatinine ratio (UCa/Cr) in male dogs of this breed, with the goal of improving the utility of the UCa/Cr.

**Hypotheses:**

(1) Significant differences will exist in preprandial and postprandial UCa/Cr between CaOx urolith‐forming and control Schnauzers. (2) The UCa/Cr will increase significantly from the first morning baseline at ≥1 postprandial time point(s) in both control and CaOx urolith‐forming dogs. (3) Biochemical abnormalities and other variables may be associated with urolith status.

**Animals:**

Twenty‐four male Miniature Schnauzer dogs, consisting of 9 with (urolith formers) and 15 without (controls) CaOx uroliths.

**Methods:**

Urine was collected before and 1, 2, 4, and 8 hours after feeding a standardized diet. Receiver operator characteristic curve analysis was performed to identify the UCa/Cr cutoff that most accurately differentiates dogs based on urolith status.

**Results:**

Urolith formers had significantly higher mean UCa/Cr over the course of 8 hours. The postprandial change in UCa/Cr was not significant at any time point between or within groups. The cutoff UCa/Cr value of 0.06 had a specificity of 93% (95% confidence interval [CI], 80%‐100%) and a sensitivity of 56% (95% CI, 21%‐86%) for identifying CaOx urolithiasis.

**Conclusions and Clinical Importance:**

Urolith‐forming male Miniature Schnauzers have excessive calciuresis, and the postprandial sampling time up to 8 hours is not critical. This simple urine measurement has potential as a marker of CaOx disease.

**Abbreviations**
CaOx: calcium oxalate
CI: confidence interval
DER: daily energy requirement
ROC: receiver operating characteristic
SSA: sulfosalicylic acid precipitation
VTH: Veterinary Teaching Hospital
UCa/Cr: urinary calcium to creatinine ratio

## 1 INTRODUCTION

Calcium oxalate (CaOx) urolithiasis can be detected in approximately 26% of older, healthy dogs of high‐risk breeds such as the Miniature Schnauzer.^1^ Forty‐eight percent of dogs will have recurrence within 3 years,^2^ which may be a consequence of limited understanding of CaOx pathogenesis and a failure to address the underlying cause. Identifying a marker of the disease will help better assess the risk of initial CaOx formation or recurrence.

Urine composition from dogs with CaOx urolithiasis differs from healthy dogs. Relative supersaturation^3^ and urine calcium concentration^1^, ^3^, ^4^ appear to be the most consistent differences. Of these, measurement of urinary calcium concentration, as a measure of urine calcium excretion (calciuresis), is more cost‐effective and readily available. Although calciuresis traditionally has been assessed by performing 24‐hour urine collection, convenience can be improved by performing a spot measurement. To account for variations in urine water content throughout the day, urine calcium concentration can be indexed to concurrent urine creatinine concentration (UCa/Cr). In healthy children, the UCa/Cr correlates moderately well with 24‐hour urinary calcium measurements in some studies.^5^, ^6^ Although this correlation has not been investigated in dogs, UCa/Cr has the potential to be a convenient alternative for estimating calciuresis in dogs.

No accepted reference range for UCa/Cr in dogs exists, but a recent study showed a significant difference in UCa/Cr between preprandial control and urocystolith‐forming dogs of 3 different breeds.^1^ Therefore, a goal of our study was to assess the UCa/Cr as a diagnostic tool to identify CaOx urolith disease and determine the most accurate cutoff value. Furthermore, previous data also shows that 6 CaOx urolith‐forming dogs had increased postprandial calciuresis compared to their calcium excretion in preprandial state.^4^ A better understanding of variation in calciuresis with regard to preprandial and postprandial states could improve the specificity of the UCa/Cr to differentiate between control and CaOx urolith‐forming dogs. If postprandial urine calcium excretion differs between CaOx urolith‐forming and non‐CaOx‐urolith forming dogs, this difference could be utilized to improve the discriminatory power of the UCa/Cr. A variety of other patient variables, including sex, breed, diet, concurrent diseases and medications, have been associated with CaOx urolith formation.^7^ Any attempt to investigate the effect of postprandial calciuresis should attempt to control for these variable as much as possible.

Our specific aims were firstly to quantify differences in the first‐morning baseline and postprandial UCa/Cr between urolith‐forming (as defined by nephroliths, urocystoliths, or both) and control male Miniature Schnauzers. The second aim was to compare the UCa/Cr in the postprandial period to baseline UCa/Cr within and between urolith‐forming and control male Miniature Schnauzers.

## 2 MATERIALS AND METHODS

### 2.1 Case selection

The study was advertised to clients of the Veterinary Teaching Hospital (VTH) at the Virginia‐Maryland College of Veterinary Medicine and to the local community. The experimental protocol was reviewed and approved by the Institutional Animal Care and Use Committee of Virginia Tech. Inclusion criteria were adult (>24 months of age), male (either neutered or intact), Miniature Schnauzer dogs. Dogs with diseases that alter calcium excretion (eg, hypercalcemia, hyperparathyroidism, renal disease, diabetes mellitus, osteolytic disease, granulomatous disease, and hyperadrenocorticism) were excluded. To identify concurrent disease, the medical history was reviewed, in addition to performing a physical examination, serum biochemistry profile, urinalysis, abdominal radiography, and urinary tract ultrasonography. Body condition score (1‐9) was recorded. A diet history was collected for each subject. Dogs were excluded if they were not consuming a complete and balanced commercial adult maintenance dog food, substantiated by Association of American Feed Control Officials nutrient profiles or feeding test, as the main portion of their diet. Furthermore, dogs were excluded if they were consuming a commercial prescription veterinary urinary dog food because such a diet would be expected to alter urine constituents. Current diets were reviewed by a board‐certified veterinary nutritionist. Dogs currently receiving medications that are known to alter calcium excretion (glucocorticoids, furosemide, thiazide diuretics, levothyroxine, theophylline, or potassium citrate) also were excluded. Dogs were prospectively enrolled with the informed owner consent.

Dogs were classified into 2 groups based on client history, medical records review, and results of abdominal radiography and urinary tract ultrasonography (kidneys, ureters, bladder) performed by the investigators. The groups were (1) urolith former dogs with previously removed or currently present CaOx uroliths anywhere in their urinary system or (2) breed‐matched controls with no history or current presence of uroliths.

If urolithiasis was identified by either diagnostic imaging or the medical history, the following criteria then were applied to determine if it was reasonable to conclude that uroliths were composed of CaOx. The criteria were either confirmatory CaOx urolith analysis or neutral or acidic urine with the lack of evidence of bacterial urinary tract infection, hepatic disease, or crystalluria of any type other than CaOx. Dogs concluded to have other types of uroliths were excluded from the study.

### 2.2 Study procedures

Dogs were presented to the VTH the day before sample collection. The daily energy requirement (DER) was calculated using the equation 1.6 × 70 × BW^0.75^, where BW is the current body weight in kilograms. All dogs were fed a standardized diet (Science Diet Adult 1‐6 years Chicken and Barley Entrée Canned Dog Food; Hills Pet Nutrition, Inc, Topeka, Kansas) to meet the DER, divided into evening and morning meals, 12 hours apart. Dogs had to eat at least half of each prescribed meal to remain in the study. Dogs were walked 3‐5 hours after the evening meal to ensure they urinated. The next morning, an initial urine sample was collected before feeding (first‐morning or baseline). After the baseline sample was collected, dogs were fed their morning meal. Urine was collected at 4 postprandial time points (referred to as the 1‐, 2‐, 4‐, and 8‐hour postprandial samples).

Urine was collected by voiding whenever possible, but if dogs did not voluntarily urinate, a sample was collected by aseptic urinary catheterization or cystocentesis, whichever was least stressful for individual dogs.

### 2.3 Laboratory measurements

Initial preprandial blood and urine samples were submitted immediately upon collection to the VITALS Laboratory of the Virginia Maryland College of Veterinary Medicine for a serum biochemistry profile and urinalysis. If the urine dipstick detected ≤2+ proteinuria, then a sulfosalicylic acid precipitation (SSA) test was performed. If this test was also positive, then a urine protein to creatinine ratio was calculated. If the serum total calcium concentration was outside the laboratory reference range, then a serum ionized calcium concentration was determined.

The baseline and postprandial urine samples were submitted immediately for calcium and creatinine measurements. Urine calcium and creatinine concentrations were measured by spectroscopy on all 5 samples using a Beckman Coulter AU 480 analyzer, using the calcium‐sensitive Arsenazo dye and the modified Jaffe procedures, respectively.

### 2.4 Statistical analysis

The primary outcome was UCa/Cr, whereas the primary exposure of interest was urolith status (urolith former versus control). A power analysis using the analysis of variance (ANOVA) of PASS^8^ and previously published data on variation of calciuresis in healthy and urolith‐forming Miniature Schauzers^4^ showed that 11 dogs per group would need to detect a difference between cases and controls with a power of 83%. An initial tentative UCa/Cr reference range for Miniature Schnauzer dogs was extrapolated from the previously published literature.^1^ This range was based on the UCa/Cr interquartile range from Miniature Schnauzers with no history of urolithiasis and yielded 0.05 as the upper limit of normal. Dogs were classed as hypercalciuric if their mean UCa/Cr using all time points was above this proposed cutoff value.

To estimate the difference in UCa/Cr based on urolith status, UCa/Cr least square means for urolith‐formers versus controls were compared using the mixed‐model ANOVA with samples from all time points. To assess for postprandial change, firstly ΔUCa/Cr was calculated for each dog at each time point using [UCa/Cr_*x*_] − [UCa/Cr_0_], where UCa/Cr_0_ is the baseline of the individual dog, and *x* refers to the specific postprandial time point. Secondly the %ΔUCa/Cr was calculated using the formula ([ΔUCa/Cr]/[UCa/Cr_0_]) × 100.

Several dog variables including age, body condition, proteinuria, hypertriglyceridemia, and hypercholesterolemia were selected for further analysis of their potential effect on urolith status, based on both previously published associations with CaOx urolith formation and preliminary inspection of the data.^1^, ^7^, ^9^

Normal probability plots showed that UCa/Cr, ΔUCa/Cr, %ΔUCa/Cr, age, and serum cholestrerol concentration followed a normal distribution, whereas body condition score and serum triglyceride concentration were skewed. Normally distributed variables were summarized as means (standard deviation) and skewed variables as medians (range). Categorical variables were summarized as contingency tables. Associations between urolith status and potential confounders were assessed using 2‐sample *t*‐tests (age and cholesterol), Wilcoxon rank sum test (body condition score and triglycerides) and Fisher's exact test (presence of proteinuria, presence of hypercalciuria). A mixed‐model ANOVA was used to assess the impact of urolith status (urolith former versus control) and time (first morning versus 1‐, 2‐, 4‐, 8‐hour postprandial) on UCa/Cr, ΔUCa/Cr, and %ΔUCa/Cr (separately). Each model also specified dog identification as a random effect, and residuals were inspected to verify that the errors were normally distributed with a mean of zero and a constant variance.

Age, proteinuria, cholesterol, and triglycerides were added to each of the mixed‐model ANOVAs but none reached statistical significance (data not shown). A receiver operating characteristic (ROC) curve analysis was performed to determine the most appropriate threshold for using the UCa/Cr to identify CaOx urolithiasis in male Miniature Schnauzers. For the ROC curve analysis, the mean UCa/Cr for each dog was calculated. Based on this result, an ROC was constructed to determine the ideal UCa/Cr cutoff value that yielded maximum sensitivity and specificity for differentiating urolith formers from control dogs. Additionally, logistic regression was used to compute an odds ratio with a 95% confidence interval (CI) for the association between calciuresis (as a risk factor) and urolith formation (as the outcome).

Statistical significance was set at *P* < .05. Analyses were performed using SAS 9.4 and JMP Pro 14.0 (ROC analysis only; The SAS Institute Inc, Cary, North Carolina).

## 3 RESULTS

Twenty‐four male Miniature Schnauzers were enrolled: 9 urolith formers and 15 healthy controls. The urolith formers consisted of 2 dogs with nephroliths only, 1 dog with urocystoliths only, 5 dogs with nephroliths and urocystoliths, and 1 dog with nephroliths and a history of urocystolith removal 2 years before. Comparative data for the 2 groups of dogs are presented in Table 1. The urolith formers were older, although this difference was not significant. Dogs were predominately neutered, with the exception of 1 intact male in each group. Both groups had similar body weights and body condition scores (mean, 5.6/9). Proteinuria, as detected by the SSA test, was identified in 20% of control dogs and 44% of urolith formers, but this difference was not significant. Mean urine protein to creatinine ratio was higher in the 4 urolith formers than in the 2 controls in which it was measured, but this difference was not significant. Serum cholesterol and triglyceride concentrations were both significantly higher in the urolith formers than in the controls.

**Table 1:** Comparative data for 15 control and 9 urolith‐forming Miniature Schnauzers

	Controls	Urolith‐formers	*P*‐value
Age (years)*	7.2 (±3.7)	9.4 (±2.0)	.11
Body Condition Score (/9) #	6.0 (±1.0)	5.0 (±1.0)	1.00
Serum triglycerides (mg/dL) #	98.0 (±147.0)	451.0 (±516.0)	**.008**
Serum cholesterol (mg/dL)*^a^	210.1 (±39.6)	273.6 (±93.5)	**.03**
Presence of proteinuria	3/15	4/9	.36
UP:C#	1.1 (±1.43) (n = 2)	2.5 (±0.94) (n = 4)	.30
UCa/Cr*	0.035	0.061	**.02**
0	0.034 (±0.018)	0.052 (±0.031)	.13
1 hour	0.031 (±0.017)	0.063 (±0.036)	**.01**
2 hours	0.039 (±0.024)	0.061 (±0.027)	.07
4 hours	0.042 (±0.039)	0.064 (±0.035)	.08
8 hours	0.044 (±0.017)	0.064 (±0.036)	**.001**
% *Δ*UCa/Cr *			
1 hour	−2.14 (±38.8)	42.9 (±77.1)	.12
2 hours	19.8 (±49.3)	45.2 (±71.1)	.38
4 hours	32.9 (±80.4)	45.9 (±65.7)	.65
8 hours	36.3 (±83.9)	49.4 (±72.7)	.65
Presence of hypercalciuresis (UCa/Cr >0.05)	2/15	6/9	**.02**

*Note*: Variables of control and urolith‐former dogs. Normally distributed variables are marked by an asterisk (*); the mean values are listed and the standard deviation in parentheses. Skewed variables are marked by a pound (#) with the median listed, and the interquartile range in parentheses. Significant *P*‐values are shown in bold font. Hypercalciuresis was initially defined as UCa/Cr >0.05 based on Furrow et al.^1^

Abbreviations: UCa/Cr, urinary calcium to creatinine ratio; UP:C, urine protein to creatinine ratio.

a Three patients in the control group were not in a fasted state at the time of sampling.

All the dogs consumed at least half of their calculated ration, with the majority of dogs voluntarily consuming the entire meal. All urine samples except 1 were voided samples. The remaining sample was collected by ultrasound‐guided cystocentesis, which included emptying the bladder as much possible during sample collection. Across all 120 urine samples collected, the urolith formers had significantly higher UCa/Cr compared to controls (least square means, 0.061 versus 0.035; *P* = .02). A dog that had hypercalciuria (defined as UCa/Cr > 0.05) had 13 times the odds (95% CI, 2‐99) of being a urolith former than did a control dog when using urine samples from all 5 times.

**Figure 1:**
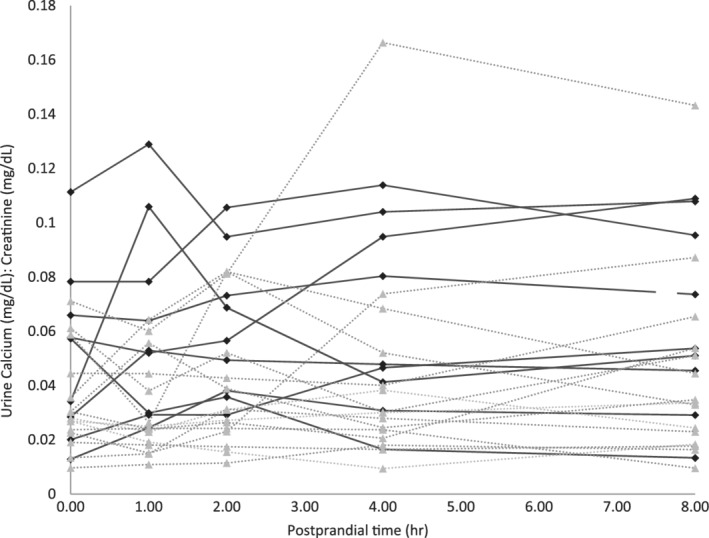
Urinary calcium to creatinine ratio values throughout the study period. Each line represents one dog, either urolith former (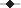) or control (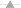)

**Figure 2:**
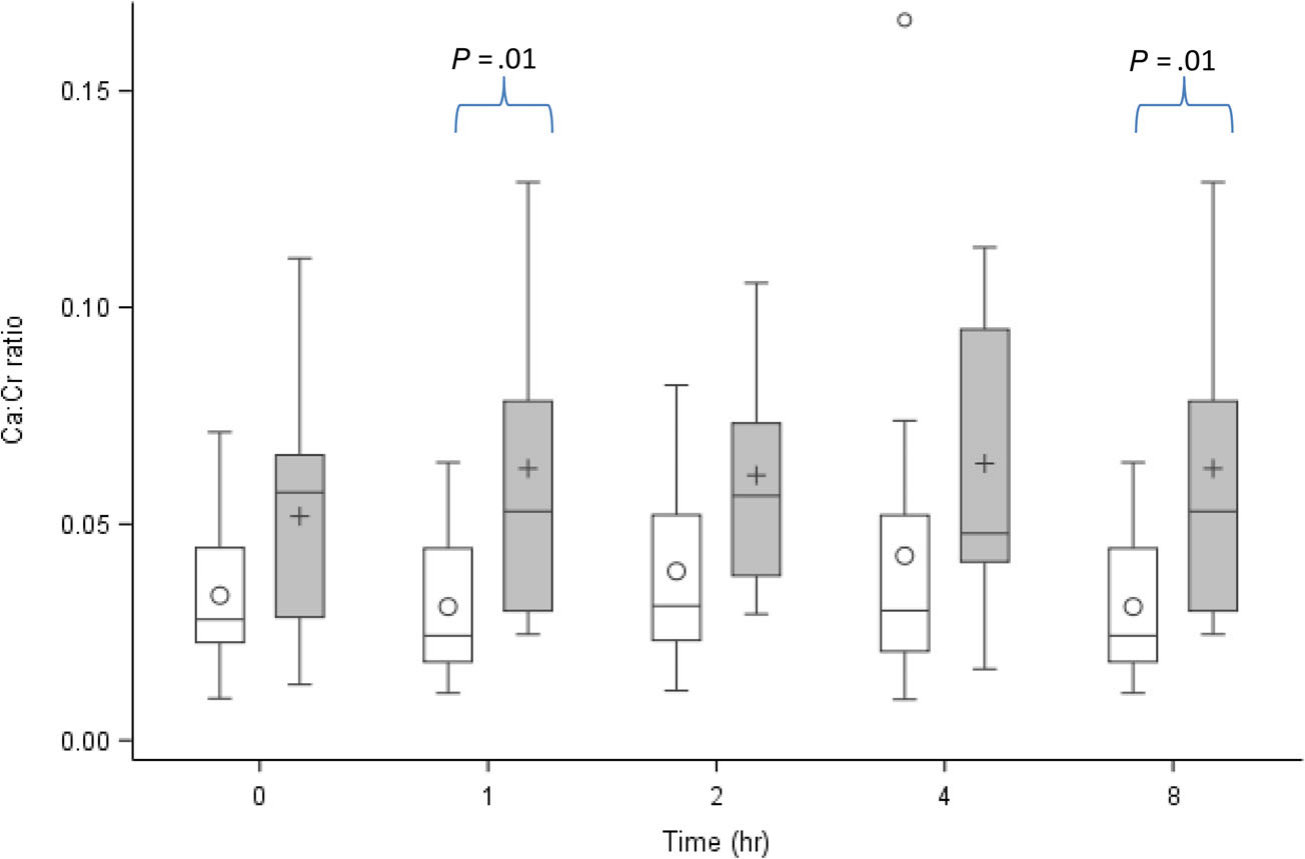
Urinary calcium to creatinine ratio at different postprandial time points in control (white boxes) and urolith‐forming (gray boxes) Miniature Schnauzer dogs. The box represents the median and the IQR, the whiskers represent values within 1.5 IQR. Circles and crosses within the box represent mean values. Values outside 1.5 IQR were deemed outliers, represented by circles and/or crosses outside the box. IQR, interquartile range

**Figure 3:**
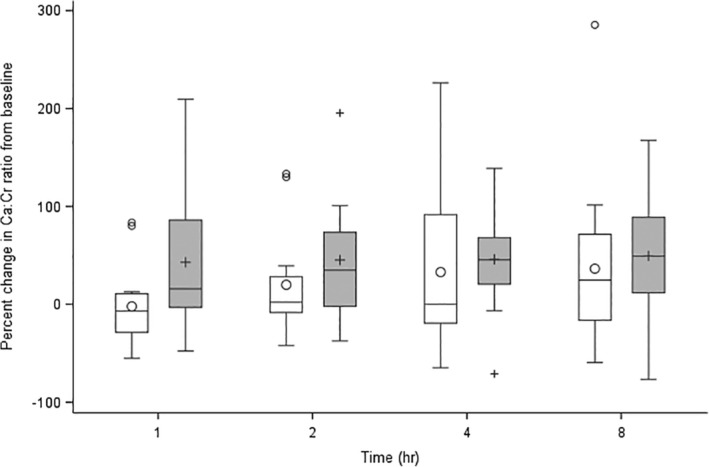
Box plot showing % *Δ*UCa/Cr for control dogs (white boxes) and urolith‐formers (gray boxes). The box and line represent the IQR and median, respectively. The circles and crosses within boxes represent the mean. The whiskers represent values within 1.5 IQR. Values outside 1.5 IQR are considered outliers and represented either circles or crosses outside boxes. There are no significant changes between the groups at any time point. IQR, interquartile range; *Δ*UCa/Cr, change in urinary calcium to creatinine ratio

When examining specific time points, urolith formers had significantly higher UCa/Cr compared to controls at 1 and 8 hours postprandial (Figure 1). Individual UCa/Cr results are presented in Table 1. A significant postprandial effect on UCa/Cr was not identified in either group. The ΔUCa/Cr at 1, 2, 4, and 8 hours postprandial were not significantly different from zero, and the ΔUCa/Cr was not significantly different between the groups. The %ΔUCa/Cr also were not significantly different from zero or between urolith‐forming and control groups (Figures 2 and 3).

Based on the ROC analysis of this population, an optimal UCa/Cr cutoff of 0.060 was determined (area under the curve, 0.748; standard error, 0.107). With this cutoff value, the UCa/Cr had a sensitivity of 56% (95% CI, 21%‐86%) and a specificity of 93% (95% CI, 80%‐100%) for detecting CaOx urolithiasis in male Miniature Schnauzers. Furthermore, the positive predictive value of a UCa/Cr >0.06 was 83% (95% CI, 53%‐100%) and the negative predictive value of a UCa/Cr <0.06 was 78% (95% CI, 59%‐97%).

## 4 DISCUSSION

We confirmed an association of UCa/Cr with CaOx urolith status in male Miniature Schnauzers, which provides further evidence that abnormal calciuresis is a mechanism underlying CaOx urolithiasis in this population.

Significant increases in postprandial UCa/Cr were not identified, which is contrary to previous observations of postprandial calciuresis measured by the 24‐hour urine collection.^4^ Our study does not necessarily exclude postprandial calciuresis, but rather we conclude that the UCa/Cr in a clinically relevant model is not a sufficiently sensitive tool to detect it. Normalizing urine calcium concentration, or any other analyte, to urine creatinine concentration will only lead to a significant linear relationship with a 24‐hour excretion if both urine calcium and creatinine concentrations remain steady or fluctuate in unison throughout a 24‐hour period. Further studies of UCa/Cr compared with the 24‐hour urine excretion in dogs are needed to determine the correlation coefficient. Likewise, studies in human populations have shown variably significant relationships between spot UCa/Cr and 24‐hour urine calcium quantification.^6^, ^10^, ^11^ Although 24‐hour urine collection is needed to accurately identify subtle postprandial calciuresis, our goal was to investigate a practical measurement to be utilized by veterinarians in clinical practice to identify calciuresis and possibly predict urolith status. It may have been possible to identify a difference if dogs had a longer fasting period such as 24 hours as used in the previous studies.^4^ Likewise, it may have been possible to identify a postprandial difference in UCa/Cr by feeding an extremely high calcium diet (ie, calcium loading). However, we elected to investigate the effects of a balanced diet for adult dogs, which was expected to be more similar to the regular diet of most dogs. Based on our study, the impact of food consumption on interpreting the UCa/Cr is negligible.

Urine Ca/Cr was sensitive enough to detect a significant difference in calciuresis between the affected and control dogs. Although significant differences were identified only at 1 and 8 hours postprandially, these specific time points are unlikely to reflect a clinically relevant difference from other time points. When examining all individual dog results (Figure 1), many urolith‐forming Miniature Schnauzers had hypercalciuria (initially defined as mean UCa/Cr > 0.05) throughout the day. Prolonged urine supersaturation may be a mechanism underlying CaOx urolithiasis in Miniature Schnauzers. When using the UCa/Cr to estimate calciuresis, the time of sample collection does not appear to be critical.

When using the UCa/Cr as an indicator of CaOx urolith formation, we propose a tentative cutoff of 0.06. The specificity and sensitivity reported here are based on the mean UCa/Cr, and more variability would be introduced if a single urine sample per dog was analyzed because small fluctuations occur throughout the day as illustrated in Figure 1. However, based on our measurements, the maximum UCa/Cr value has very good specificity, because only 7% of non‐urolith‐forming Miniature Schnauzers were above this limit. This preliminary cutoff value for UCa/Cr, based on the limited numbers reported here, can be the basis for larger epidemiological studies to better define normal calciuresis and expand its utility. Because this test is noninvasive and inexpensive to perform, it could easily be added to the routine urine testing as an additional screening test for at‐risk dogs such as Miniature Schnauzers. For maximum benefit, an ideal screening test should detect disease in the early stages. Although it seems reasonable that increased urinary calcium concentrations would occur before formation of clinically detectable uroliths, long‐term studies monitoring UCa/Cr and the subsequent development of urolithiasis should be performed to confirm this supposition.

The UCa/Cr cutoff value of 0.06 has only modest sensitivity because it could be expected to correctly detect 56% of urolith formers based on the population of dogs included in our study. The remaining 44% of urolith formers may have had fluctuating hypercalciuria that was not detected on the day of the study. The intra‐individual day‐to‐day variability of calcium excretion in dogs has not been studied to our knowledge. Alternatively, the urolith formers that did not have detectable hypercalciuria may have factors other than urinary calcium concentration contributing to CaOx urolith formation. Promotors and inhibitors of urinary crystal initiation, aggregation, and growth that are important for the development of CaOx urolithiasis in humans likely also contribute to the development of CaOx urolithiasis in dogs.

We did not identify proteinuria as significantly associated with CaOx urolithiasis, which is in contrast to previous findings^9^ and likely a consequence of our limited sample size. The association of CaOx urolithiasis with dyslipidemia was an interesting finding, but the influence of other confounding variables such as older age in the urolith former group has not been eliminated. Humans with metabolic syndrome typically have hypercholesterolemia and hypertriglyceridemia, which are associated with subepithelial tubular damage, development of Randall's plaques, crystal formation, and nephrolithiasis.^12^ It is not known if nephroliths form by the same mechanisms in dyslipidemic dogs, but it is an interesting possibility given the association of these comorbidities in our cohort. Alternatively, dyslipidemias, proteinuria, and CaOx urolithiasis may be common but unrelated conditions in older Miniature Schnauzers.

Although it would not have been appropriate to do with the small population included in our study, regression analysis in larger studies could be applied to remove confounding variables such as age. An additional strategy would be to select age‐matched controls to account for the effect of this potentially important variable on calcium excretion. Larger studies also would allow regression analysis to identify and better quantify contributions from other variables such as proteinuria, hypercholesterolemia, and hypertriglyceridemia.

Dietary composition has been examined previously for its role in urolith formation.^13^ In our study, comparing current or previous diets between urolith status groups or calciuresis groups could not be done because of the wide variety of commercial dog foods fed. Dogs were fed commercial dry or canned foods or both, which may have influenced total dietary moisture intake. We attempted to minimize the effect of diet variation by feeding a standardized diet 1 meal before and after the first morning sample.

Our study had several other limitations. First, dogs were classified based on the predicted urolith type. Not all dogs had urolith analysis information available, and it is possible that dogs that were predicted to have CaOx uroliths had a different type of urolith.

Second, dogs were given ample opportunity to empty their bladders at each time point, but residual urine volumes were not measured. Therefore, it is possible that some urine produced during the previous time period may have remained in the bladder and confounded subsequent results.

Third, it is possible that some control dogs may develop urolithiasis in the future. The mean age of dogs diagnosed with CaOx urolithiasis is reported to be 8 years,^7^ and thus younger control animals may be latent urolith formers. It is not known if the UCa/Cr would increase before the onset of the clinical disease, although such a finding would be useful to proactively manage high‐risk dogs. Ideally, control dogs could be followed over time to determine if the UCa/Cr can identify future urolith formers before the onset of clinically detectable urolithiasis. In addition, following the UCa/Cr of urolith formers over time could be used to help identify the effectiveness of prevention measures designed to decrease calciuresis. Doing so would be considerably more convenient for investigators than measuring 24‐hour urine calcium excretion, such as performed previously to assess the impact of hydrochlorothiazide.^14^

In conclusion, hypercalciuria is common among male urolith‐forming Miniature Schnauzers. The UCa/Cr proved to be a simple and inexpensive test that has the potential to be used to identify dogs at risk for CaOx urolithiasis. A tentative cutoff or upper limit of normal UCa/Cr is 0.06; 93% of male Miniature Schnauzers with a UCa/Cr >0.06 were urolith formers. Hypercalciuria appears to occur throughout the day and thus strict timing of urine collection likely is not required.

## CONFLICT OF INTEREST DECLARATION

Authors declare no conflict of interest.

## OFF‐LABEL ANTIMICROBIAL DECLARATION

Authors declare no off‐label use of antimicrobials.

## INSTITUTIONAL ANIMAL CARE AND USE COMMITTEE (IACUC) OR OTHER APPROVAL DECLARATION

Approved by the Virginia Tech IACUC, application number 16‐059.

## HUMAN ETHICS APPROVAL DECLARATION

Authors declare human ethics approval was not needed for this study.
